# Compendium of the Reactions of H_3_O^+^ With Selected Ketones of Relevance to Breath Analysis Using Proton Transfer Reaction Mass Spectrometry

**DOI:** 10.3389/fchem.2019.00401

**Published:** 2019-06-13

**Authors:** Michaela Malásková, David Olivenza-León, Felix Piel, Paweł Mochalski, Philipp Sulzer, Simone Jürschik, Chris A. Mayhew, Tilmann D. Märk

**Affiliations:** ^1^Institute for Breath Research, Fakultät für Chemie und Pharmazie, Leopold-Franzens-Universität Innsbruck, Dornbirn, Austria; ^2^Molecular Physics Group, School of Physics and Astronomy, University of Birmingham, Birmingham, United Kingdom; ^3^IONICON Analytik Gesellschaft m.b.H., Innsbruck, Austria; ^4^Institut für Ionenphysik und Angewandte Physik, Universität Innsbruck, Innsbruck, Austria; ^5^Institute of Chemistry, Faculty of Mathematics and Natural Sciences, Jan Kochanowski University, Kielce, Poland

**Keywords:** ketones, breath analysis, PTR-MS, reduced electric field, fastGC

## Abstract

Soft chemical ionization mass spectrometric techniques, such as proton transfer reaction mass spectrometry (PTR-MS), are often used in breath analysis, being particularly powerful for real-time measurements. To ascertain the type and concentration of volatiles in exhaled breath clearly assignable product ions resulting from these volatiles need to be determined. This is difficult for compounds where isomers are common, and one important class of breath volatiles where this occurs are ketones. Here we present a series of extensive measurements on the reactions of H_3_O^+^ with a selection of ketones using PTR-MS. Of particular interest is to determine if ketone isomers can be distinguished without the need for pre-separation by manipulating the ion chemistry through changes in the reduced electric field. An additional issue for breath analysis is that the product ion distributions for these breath volatiles are usually determined from direct PTR-MS measurements of the compounds under the normal operating conditions of the instruments. Generally, no account is made for the effects on the ion-molecule reactions by the introduction of humid air samples or increased CO_2_ concentrations into the drift tubes of these analytical devices resulting from breath. Therefore, another motivation of this study is to determine the effects, if any, on the product ion distributions under the humid conditions associated with breath sampling. However, the ultimate objective for this study is to provide a valuable database of use to other researchers in the field of breath analysis to aid in analysis and quantification of trace amounts of ketones in human breath. Here we present a comprehensive compendium of the product ion distributions as a function of the reduced electric field for the reactions of H_3_O^+^. (H_2_O)_n_ (*n* = 0 and 1) with nineteen ketones under normal and humid (100% relative humidity for 37 °C) PTR-MS conditions. The ketones selected for inclusion in this compendium are (in order of increasing molecular weight): 2-butanone; 2-pentanone; 3-pentanone; 2-hexanone; 3-hexanone; 2-heptanone; 3-heptanone; 4-heptanone; 3-octanone; 2-nonanone; 3-nonanone; 2-decanone; 3-decanone; cyclohexanone; 3-methyl-2-butanone; 3-methyl-2-pentanone; 2-methyl-3-pentanone; 2-methyl-3-hexanone; and 2-methyl-3-heptanone.

## Introduction

Depending on the actual mass resolution, current proton transfer reaction mass spectrometers (PTR-MS) are easily capable of separating many protonated isobaric compounds through a peak fitting procedure providing their mass separation is at least 0.01 Da. The selectivity of PTR-MS can be further improved by the manipulation of the ion-molecule chemistry that occurs between a reagent ion and a given isobar in the drift tube to produce different product ions. This can be achieved by (i) changing the reagent ion, examples for which have been presented in the literature for explosives (Sulzer et al., 2013; Agarwal et al., 2014), or psychoactive substances (Acton et al., 2014; Lanza et al., 2015), and/or (ii) the collisional processes in the drift tube through changing the reduced electric field. Changes in the reduced electric field (the ratio of the electric field strength, *E*, to the gas number density, *N*, in the drift tube) to alter the product ion distributions have been demonstrated in the areas of homeland security, e.g., detection of chemical warfare agents (Petersson et al., 2009), explosives (Mayhew et al., 2010; Sulzer et al., 2012, 2013), and rape drugs (Jürschik et al., 2012), and in environmental science, e.g., the identification of monoterpenes (Materić et al., 2017).

This application of changing collisional processes through changes in the reduced electric field to enhance compound selectivity has led to the development of a computer- controlled fast switching drift tube voltage (González-Méndez et al., 2018) and the adaptation of a radio frequency ion-funnel drift tube (González-Méndez et al., 2016).

Although today there are several ways to enhance the selectivity of PTR-MS for isobaric compounds, distinguishing isomeric compounds is still more of an issue. With no pre-separation of isomeric compounds, rarely can isomers be easily identified using PTR-MS through differences in product ion distributions, even if the ion-molecule chemistry occurring in the drift tube of PTR-MS is manipulated in a structured way. One study has demonstrated how reactions of ${\text{O}}_{2}^{+}$ and NO^+^ can be used to distinguish two isomeric mephedrone substitutes (4-methylethcathinone and N-ethylbuphedrone) whereas reactions with H_3_O^+^ could not (Lanza et al., 2013). However, such examples in ion-isomer chemistry are usually the exception rather than the rule.

Isomers of ketones are so far difficult to identify unambiguously with a PTR-MS instrument. Pre-separation offered by standard gas chromatography (GC) techniques can be used, but they take away the main advantage of PTR-MS, namely its real-time analytical capabilities. The recent development of fast gas chromatography (fastGC) coupled to PTR-MS provides a compromise between real-time measurements, ensuring reasonably fast analysis (within approximately 90 s), whilst still taking advantage of limited pre-separation of compounds to improve the analytical specificity of PTR-MS (Ruzsanyi et al., 2013; Romano et al., 2014; Anderson, 2015).

In this paper we have used the fastGC PTR-MS technique in order to accurately determine the product ion distributions for a large selection of ketones as a function of reduced electric field so that we can unambiguously determine their product ions, without any concerns from impurities in the samples. Ketones have been selected for this study, because they form a common class of compounds found in breath, blood and urine (de Lacy Costello et al., 2014), and their detection holds many possibilities for non-invasive diagnostic and monitoring procedures in health services. One example is the diagnosis of ketosis, resulting from the elevation of ketone bodies in the blood. Detecting changes in ketone concentrations could thus be used to diagnose diabetic ketosis. A key ketone found in high concentrations in breath is acetone, the production of which (as for most ketones) is linked to fat metabolism, and hence its detection in breath could provide a window to predict fat loss (Anderson, 2015). Given the importance of acetone in the breath, it has been investigated numerous times with PTR-MS, and hence acetone does not form part of this current study. Less attention has been given to other ketones in PTR-MS studies. Hence this investigation has focused its attention on other important breath ketones, although generally found in much lower concentrations in the breath than for acetone. This has produced a wealth of new data, providing a useful database of the product ion distributions resulting from the reactions of H_3_O^+^ and H_3_O^+^. (H_2_O) with ketones using PTR-MS.

Awareness of possible changes in the reaction processes occurring in the drift tube of a PTR-MS instrument resulting from changes in humidity have been known for some time (Warneke et al., 2001; Tani et al., 2003, 2004). Breath samples are humid, and thus product ion distributions determined under the “normal” operating conditions of PTR-MS (e.g., using purified air or nitrogen as the buffer gas in the drift tube) may not be a true reflection of those associated with a humid gas sample in the drift tube. This is because it can be expected that a higher humidity associated with breath samples (100% relative humidity at 32–34°C) will affect the product ion distributions through changes in the energy associated with the collisional processes. Furthermore, if protonated water clusters can react with a breath volatile via proton transfer, far less energy will be available in the reaction than for that associated with H_3_O^+^, and with higher humidity comes a greater production of protonated water clusters for a given reduced electric field. This effect will generally be more important at low reduced electric field values (i.e., < ~120 Td, 1 Td = 10^−17^ V cm^2^) when collisions will lead to less break-up of the protonated water clusters to H_3_O^+^ and neutral water(s). Moreover, if the protonated water clusters cannot react with a volatile, then a reduction in the sensitivity of detection of that compound results. Finally, differences in product ion distributions will arise if secondary processed occur, such as when primary product ions react with water.

A review of the literature shows that PTR-MS product ion distributions of compounds of interest to breath research are generally determined under the “normal” operating conditions, i.e., where the humidity in the drift tube is determined by the diffusion of water from the discharge region into the drift tube, which will be less than that associated with a breath sample. An objective of this work is to improve our knowledge on the effects of humidity on product ion distributions.

The first studies associated with investigating the effects of humidity on reaction processes in PTR-MS focused on sensitivity issues. For example, Warneke et al. (2001) showed how the sensitivity for the detection of benzene and toluene at fixed reduced electric fields decreased with increasing humidity, owing to unreactive H_3_O^+^.(H_2_O)_n_ clusters. Hence, de Gouw et al. (2003) suggested employing a humidity factor to determine the concentrations of a compound if it reacts with protonated water clusters, a factor which takes into account the efficiencies of the proton transfer reaction and the transmission of H_3_O^+^.(H_2_O) relative to that of H_3_O^+^. These factors were determined and used to correct for the influences of humidity on the detection sensitivity for methanol, acetonitrile, acetaldehyde, acetone, benzene and toluene by de Gouw and Warneke (2007). A PTR-MS investigation of the effects of humidity on the product ion distributions resulting from the reactions of H_3_O^+^ with two sesquiterpenes (α-cedrene and longifolene) was undertaken by Demarcke et al. (2009). In that study, no substantial influence of the humidity in the drift tube on the product ion yields was observed. More recently, the effects of humidity on product ion distributions have been investigated for α-pinene, δ-limonene, and longifolene by Kari et al. (2018) at two different *E/N* values (80 Td and 130 Td) (Kari et al., 2018), and for more than 20 volatile organic compounds (VOCs), including aldehydes, ketones, aromatic compounds and hydrocarbons by Trefz et al. (2018) at one fixed *E/N* (139 Td) (Trefz et al., 2018). In the former study, no significant changes in the product ion distributions were observed. However, Trefz et al. reported large differences in VOC intensities between “dry” and “humid” samples. Thus, the effect of humidity appears to depend very much on the volatile chemical compound.

In this paper we present details on the reactions of H_3_O^+^ and associated water clusters with a selected number of ketones over a large reduced electric field range of 100–220 Td, and compare product ions obtained under “normal” and “humid” operating conditions of the drift tube. This work demonstrates that changes in product ion distributions do occur for fixed *E/N* for different humidities, and hence it clearly demonstrates that humidity effects should be considered when relying on product ion distributions for undertaking breath research with a PTR-MS.

## Materials and Methods

### Sample Preparation

Samples were prepared in two different ways depending on the humidity of the measurement.

For measurements under normal conditions, an open glass vial containing a ketone was purged with high purity N_2_ (Alphagaz 1, Air Liquide GmbH., Austria), which had been previously passed through a P300-1 Filter (VICI AG, Switzerland) for purification (6.0). The vial was then covered with parafilm. Using a glass syringe a quantity of headspace was taken from the vial through the parafilm. This headspace containing the ketone and N_2_ was then injected into a PTFE bag filled with 3L of dry 6.0 N_2_, which was already connected to the inlet of the PTR-ToF-MS instrument. This injected volume into the bag varied from 5 μL to 10 mL, depending on the volatility of the ketone.

Humid samples were prepared using a Liquid Calibration Unit (LCU, IONICON Analytik GmbH, Austria). The LCU generates defined gaseous concentrations from aqueous solutions of volatile and semi-volatile organics. A description of the LCU has already been provided in detail by Fischer et al. (2013). Briefly, a homebuilt liquid flow controller injects a defined flow into a nebuliser (X175, Burgener Research Inc., United Kingdom). Vaporization of the aqueous solution produces micro droplets, which are evaporated in a heating chamber maintained at 100°C. The heating chamber is being constantly flushed by a buffer gas, e.g., zero air or N_2_, diluting the organic sample and thus generating a continuous stream of a defined trace gas mixture.

For this study, to generate the humid samples, 16 mL glass vials, kept at a constant temperature of 30°C, were filled with a trace quantity of a ketone [1–10 μL (depending on the volatility of the ketone)] diluted in 100 mL of purified water. A sample flow of this ketone/water mixture at 35 μL/min was diluted in a N_2_ flow of 950 mL/min to achieve a 5% absolute humidity. The combined flow was then directly connected to the fastGC inlet system of the PTR-ToF-MS instrument.

For both the dry and humid measurements, the dilution of the samples were prepared to yield a concentration of the ketone in the drift tube to be approximately 100 ppbv.

The experiments presented here were done through an automated measurement procedure. This consisted of background measurements for 5 min. For the dry mixtures, this involved the PTFE bag filled only with purified N_2_. For the humid standards this step involved a vial containing only purified water. Next, the prepared samples were directed to the drift tube for a 2-min stabilization period, which was next followed by 2 min and 40 s of fastGC measurement at an *E/N* of 180 Td to help identify the product ions produced in the drift tube for a given ketone and a 26-min *E/N* set of measurements over the range 100–220 Td in steps of 10 Td (1 min each), in both directions, to provide two data sets.

### FastGC PTR-ToF-MS

**Figure 1:**
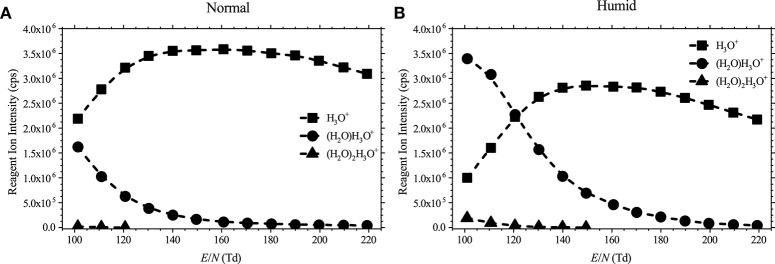
Reagent ion intensities in counts per second (cps) as a function of the reduced electric field for **(A)** normal (dry buffer gas) and **(B)** humid (5% absolute humidity buffer gas) conditions.

Details of PTR-ToF-MS and methods of operation have been reviewed extensively in the literature (Ellis and Mayhew, 2014), and therefore only brief details are required here. For this study, measurements were taken using a PTR-TOF 8000 with a fastGC add-on (IONICON Analytik GmbH, Austria) (Jordan et al., 2009; Graus et al., 2010). Briefly, water vapor is introduced into a hollow cathode discharge to generate H_3_O^+^.(H_2_O)_n_ (*n* = 0, 1, 2, …), initially through electron ionization of water and subsequent ion-molecule reactions with water. These reagent ions are then transferred to the drift tube via a focusing lens. The distribution of the protonated water clusters in the reaction region depends on the *E/N* value and the humidity in the drift tube as shown in Figure 1.

These reagent ions are then transported down the drift tube under the influence of the uniform electric field. Analytes are injected into the drift tube through an inlet pipe. Proton transfer from hydronium to the analyte takes place within the drift tube if the proton affinity (PA) of the analyte is higher than that of water (PA(H_2_O) = 691 kJ mol^−1^). Proton transfer can be non-dissociative and dissociative. However, it should be stressed that fragmentation of the protonated molecule can be a barrierless process and occur spontaneously, or it can be induced by the collision of the reagent ions with analyte and/or charged analyte with the buffer gas.

For all measurements the drift tube was kept at a pressure of 2.3 mbar, with both the inlet system and drift tube being maintained at 100°C. The collisional energies of the reagent and product ions were controlled by the value of the reduced electric field. For this study we kept the drift tube at constant pressure and temperature (and hence constant *N*), and changed the drift tube voltage to alter the value of *E*/*N*. The drift voltage could be changed from 410 V up to a maximum of 890 V. For the applied values of the drift tube pressure and temperature these values correspond to an *E/N* range from 100 to 220 Td.

Using a dry buffer gas in the drift tube of a PTR-MS instrument does not mean that it is operated under dry conditions, since some amounts of water vapor diffuse from the hollow cathode. This condition will be denoted as “normal” operating condition later in this paper. When a water saturated buffer gas was used, this is referred in the text as operating the drift tube under “humid” conditions.

FastGC was used to separate analytes of interest from possible contaminants in the produced standards. The fastGC add-on used in this study is a modification of the setup used by Romano et al. (2014) and Ruzsanyi et al. (2013). Therefore, only the modifications relevant for this study will be provided here. An MXT-1 column (10 m × 0.53 mm, film thickness 0.25 μm, dimethyl polysiloxane phase, Restek, USA) was used. The samples were injected into a 0.5 ml sample loop made of passivated stainless steel. A custom-made valve block consisting of four three-way valves and a needle valve has been replaced by a 10-port passivated valve (VICI AG, Switzerland) and a three-way gas valve made from polyether ether ketone (PEEK) was used. All parts of the inlet system are installed within the oven that houses the drift tube to prevent cold spots. This revised setup enabled constant filling of the sample loop and constant back-flushing of the capillary column with the carrier gas. 8 ml/min and 20 ml/min of 6.0 N_2_ were used as carrier gas and make-up gas, respectively. A voltage ramp of 0.5 V/s from 10 V up to 80 V was applied raising the temperature of the capillary column from room value up to 240°C.

### Chemicals

The following liquid substances were purchased from Sigma-Aldrich: 2-pentanone (98%), 3-pentanone (99%), 2-hexanone (98%), 3-hexanone (98%), 3-heptanone (analytical standard), 4-heptanone (98%), 2-nonanone (99%), 3-nonanone (99%), 2-decanone (98%), cyclohexanone (99.8%), 3-methyl-2-pentanone (99%), 2-methyl-3-pentanone (97%), 2-methyl-3-hexanone (98%), and 2-methyl-3-heptanone (99%). 2-butanone (99.5%), 2-heptanone (98.5%), and 3-methyl-2-butanone (98.5%) were purchased from Honeywell. 3-octanone (99%) and 3-decanone (97%) were purchased from Acros Organics and SAFC, respectively. These were used with no further purification.

### Data Analysis

The “PTR-MS Viewer” (IONICON Analytik GmbH, Austria) was used to identify peaks in the mass spectra and to extract peak data. Raw peak data, i.e., data not corrected for transmission factors, were normalized to 1 million reagent ions and had any backgrounds subtracted. By using the “raw” data, the product ion distributions we have determined here can be more easily compared with other measurements using different PTR-MS instruments. However, we emphasize that the product ion distributions that have been determined for the selection of ketones chosen for this study have to be taken with some caution if a PTR-TOF 8000 is not being used, and that researchers need to determine product ion distributions for their own instruments and conditions.

## Results and Discussion

Table 1 presents a summary of the product ion distributions (percentages) for all the ketones investigated in this study at three selected reduced electric fields, namely 100 Td, 140 Td, and 180 Td under normal and humid conditions. These values give a good representation of all product ions observed and quickly illustrate the effects of humidity on the product ion distributions, if any. The table starts with the thirteen linear chained ketones in order of molecular weight (MW), followed by the one cyclic ketone (cyclohexanone), and finishing with five non-linear ketones, also presented in order of increasing nominal MW, and, for low *E/N* (see Figure 1), from reactions of H_3_O^+^.H_2_O with those ketones whose proton affinities are greater than that associated with (H_2_O)_2_ (808 kJ mol^−1^), i.e., 2-butanone (827 kJ mol^−1^), 2-pentanone (833 kJ mol^−1^), 3-pentanone (837 kJ mol^−1^), and 3-methyl-2-butanone (836 kJ mol^−1^).

**Table 1:** Product ions identified and their associated product ion branching ratios (percentages) measured at reduced electric fields of 100, 140, and 180 Td resulting from the reactions of H_3_O^+^ with several ketones.

**Ketone Molecular formula Nominal MW**	**Product ion *m/z* (Th)**	**Product ion formula**	**Product ion branching percentages**					
			**Normal** ***E*****/*****N*** **(Td)**			**Humid** ***E*****/*****N*** **(Td)**		
			**100**	**140**	**180**	**100**	**140**	**180**
2-butanone	73.07	C_4_H_8_OH^+^	100	99	87	100	100	88
C_4_H_8_O	55.05	C_4_${\text{H}}_{7}^{+}$	0	1	10	0	0	10
72	39.02	C_3_${\text{H}}_{3}^{+}$	0	0	3	0	0	2
2-pentanone	87.08	C_5_H_10_OH^+^	99	67	20	99	84	29
C_5_H_10_O	45.03	C_2_H_5_O^+^	1	33	70	1	16	66
86	39.02	C_3_${\text{H}}_{3}^{+}$	0	0	10	0	0	5
3-pentanone	87.08	C_5_H_10_OH^+^	98	72	23	99	91	43
C_5_H_10_O	69.07	C_5_${\text{H}}_{9}^{+}$	1	4	2	1	4	4
86	45.03	C_2_H_5_O^+^	1	20	55	0	4	39
	41.04	C_3_${\text{H}}_{5}^{+}$	0	3	5	0	1	5
	39.02	C_3_${\text{H}}_{3}^{+}$	0	1	15	0	0	9
2-hexanone	101.10	C_6_H_12_OH^+^	100	94	48	100	95	49
C_6_H_12_O	59.05	C_3_H_7_O^+^	0	1	3	0	1	3
100	45.03	C_2_H_5_O^+^	0	5	39	0	4	40
	39.02	C_3_${\text{H}}_{3}^{+}$	0	0	10	0	0	8
3-hexanone	101.10	C_6_H_12_OH^+^	93	73	31	96	88	44
C_6_H_12_O	83.09	C_6_${\text{H}}_{11}^{+}$	1	4	4	1	4	5
100	59.05	C_3_H_7_O^+^	3	9	15	3	7	17
	55.05	C_4_${\text{H}}_{7}^{+}$	0	3	5	0	0	12
	45.03	C_2_H_5_O^+^	2	5	15	0	0	0
	41.04	C_3_${\text{H}}_{5}^{+}$	0	4	6	0	1	9
	39.02	C_3_${\text{H}}_{3}^{+}$	1	1	18	0	0	13
	31.02	CH_3_O^+^	0	1	6	0	0	0
2-heptanone	115.11	C_7_H_14_OH^+^	94	76	31	96	86	52
C_7_H_14_O	97.10	C_7_${\text{H}}_{13}^{+}$	4	10	7	2	7	9
114	59.05	C_3_H_7_O^+^	1	2	4	2	3	6
	55.05	C_4_${\text{H}}_{7}^{+}$	0	9	14	0	4	20
	45.03	C_2_H_5_O^+^	1	3	15	0	0	0
	39.02	C_3_${\text{H}}_{3}^{+}$	0	0	29	0	0	13
3-heptanone	115.11	C_7_H_14_OH^+^	98	89	35	99	95	57
C_7_H_14_O	97.10	C_7_${\text{H}}_{13}^{+}$	2	5	4	1	4	5
114	59.05	C_3_H_7_O^+^	0	0	0	0	1	7
	55.05	C_4_${\text{H}}_{7}^{+}$	0	4	8	0	0	12
	41.04	C_3_${\text{H}}_{5}^{+}$	0	1	7	0	0	3
	39.02	C_3_${\text{H}}_{3}^{+}$	0	0	27	0	0	16
	31.02	CH_3_O^+^	0	1	19	0	0	0
4-heptanone	115.11	C_7_H_14_OH^+^	98	90	52	99	95	70
C_7_H_14_O	73.07	C_4_H_9_O^+^	0	1	2	0	0	0
114	59.05	C_3_H_7_O^+^	1	2	6	1	1	4
	55.05	C_4_${\text{H}}_{7}^{+}$	0	6	15	0	4	16
	53.04	C_4_${\text{H}}_{5}^{+}$	0	0	5	0	0	3
	39.02	C_3_${\text{H}}_{3}^{+}$	1	1	20	0	0	7
3-octanone	129.13	C_8_H_16_OH^+^	99	96	46	100	98	73
C_8_H_16_O	69.07	C_5_${\text{H}}_{9}^{+}$	0	3	5	0	1	6
128	59.05	C_3_H_7_O^+^	1	1	3	0	1	4
	41.04	C_3_${\text{H}}_{5}^{+}$	0	0	11	0	0	10
	39.02	C_3_${\text{H}}_{3}^{+}$	0	0	35	0	0	7
2-nonanone	143.14	C_9_H_18_OH^+^	100	93	34	100	97	62
C_9_H_18_O	83.09	C_6_${\text{H}}_{11}^{+}$	0	0	0	0	1	4
142	69.07	C_5_${\text{H}}_{9}^{+}$	0	4	4	0	2	6
	55.05	C_4_${\text{H}}_{7}^{+}$	0	1	4	0	0	7
	41.04	C_3_${\text{H}}_{5}^{+}$	0	1	10	0	0	10
	39.02	C_3_${\text{H}}_{3}^{+}$	0	1	48	0	0	11
3-nonanone	143.14	C_9_H_18_OH^+^	100	87	48	100	100	79
C_9_H_18_O	55.05	C_4_${\text{H}}_{7}^{+}$	0	4	6	0	0	5
142	41.04	C_3_${\text{H}}_{5}^{+}$	0	8	11	0	0	6
	39.02	C_3_${\text{H}}_{3}^{+}$	0	1	35	0	0	10
2-decanone	157.16	C_10_H_20_OH^+^	100	94	48	100	99	81
C_10_H_20_O	83.09	C_6_${\text{H}}_{11}^{+}$	0	2	3	0	1	6
156	55.05	C_4_${\text{H}}_{7}^{+}$	0	3	13	0	0	13
	39.02	C_3_${\text{H}}_{3}^{+}$	0	1	36	0	0	0
3-decanone	157.16	C_10_H_20_OH^+^	99	95	48	100	100	86
C_10_H_20_O	55.05	C_4_${\text{H}}_{7}^{+}$	1	4	10	0	0	7
156	39.02	C_3_${\text{H}}_{3}^{+}$	0	1	42	0	0	7
cyclohexanone	99.08	C_6_H_10_OH^+^	99	88	30	99	93	40
C_6_H_10_O	81.07	C_6_${\text{H}}_{9}^{+}$	1	12	65	1	7	56
98	79.05	C_6_${\text{H}}_{7}^{+}$	0	0	5	0	0	3
	39.02	C_3_${\text{H}}_{3}^{+}$	0	0	0	0	0	1
3-methyl-2-butanone	87.08	C_5_H_10_OH^+^	100	98	63	99	96	66
C_5_H_10_O	69.07	C_5_${\text{H}}_{9}^{+}$	0	2	5	1	3	7
86	45.03	C_2_H_5_O^+^	0	0	8	0	0	8
	41.04	C_3_${\text{H}}_{5}^{+}$	0	0	4	0	1	8
	39.02	C_3_${\text{H}}_{3}^{+}$	0	0	20	0	0	11
3-methyl-2-pentanone	101.10	C_6_H_12_OH^+^	100	70	23	100	73	22
C_6_H_12_O	59.05	C_3_H_7_O^+^	0	11	28	0	7	26
100	57.07	C_4_${\text{H}}_{9}^{+}$	0	4	3	0	5	4
	45.03	C_2_H_5_O^+^	0	15	39	0	15	40
	39.02	C_3_${\text{H}}_{3}^{+}$	0	0	7	0	0	8
2-methyl-3-pentanone	101.10	C_6_H_12_OH^+^	98	61	17	95	74	24
C_6_H_12_O	59.05	C_3_H_7_O^+^	1	15	29	3	9	29
100	57.07	C_4_${\text{H}}_{9}^{+}$	0	4	2	0	3	3
	45.03	C_2_H_5_O^+^	1	20	41	2	13	40
	39.02	C_3_${\text{H}}_{3}^{+}$	0	0	11	0	1	4
2-methyl-3-hexanone	115.11	C_7_H_14_OH^+^	95	66	24	96	72	24
C_7_H_14_O	97.10	C_7_${\text{H}}_{13}^{+}$	5	14	8	4	10	7
114	59.05	C_3_H_7_O^+^	0	8	17	0	4	17
	55.05	C_4_${\text{H}}_{7}^{+}$	0	4	5	0	2	7
	45.03	C_2_H_5_O^+^	0	5	17	0	11	27
	41.04	C_3_${\text{H}}_{5}^{+}$	0	3	7	0	1	6
	39.02	C_3_${\text{H}}_{3}^{+}$	0	0	22	0	0	12
2-methyl-3-heptanone	129.13	C_8_H_16_OH^+^	96	76	26	97	81	28
C_8_H_16_O	111.12	C_8_${\text{H}}_{15}^{+}$	3	5	3	2	5	3
128	69.07	C_5_${\text{H}}_{9}^{+}$	0	8	5	0	4	5
	59.05	C_3_H_7_O^+^	0	0	0	0	3	14
	45.03	C_2_H_5_O^+^	0	2	8	0	3	10
	43.05	C_3_${\text{H}}_{7}^{+}$	1	2	2	1	2	3
	41.04	C_3_${\text{H}}_{5}^{+}$	0	6	15	0	2	15
	39.02	C_3_${\text{H}}_{3}^{+}$	0	1	41	0	0	22

*Values for the product ion branching percentages are given whilst operating the drift tube under “normal” conditions and under “humid” (breath humidity) conditions. Errors in the branching percentages are estimated to be <20%*.

**Figure 2:**
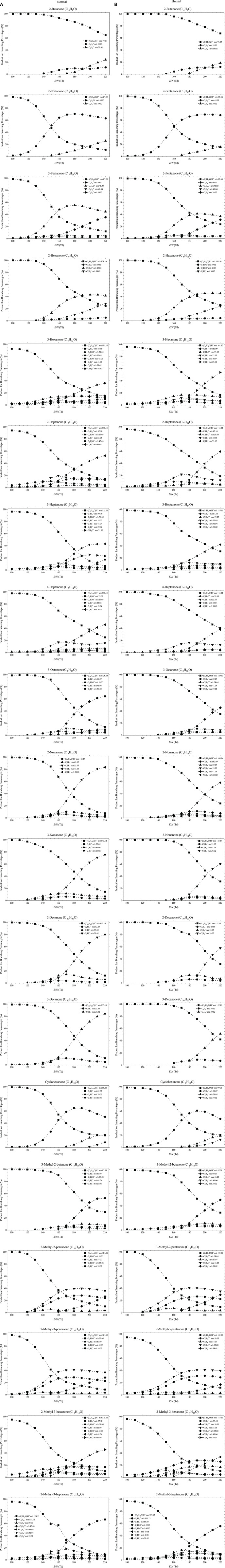
Product ion distributions (branching percentages) as a function of *E/N* resulting from reaction with H_3_O^+^ (and potentially H_3_O^+^.H_2_O as stated above) under **(A)** normal and **(B)** high humidity drift tube conditions with several ketones.

The dependence of the product ion branching percentages as a function of *E/N* are shown graphically in Figure 2. The chemical formulae of the product ions given in Table 1 and Figure 2 have been tentatively identified via the exact *m/z* (to 2 decimal places) and isotope (^13^C) intensities. Only product ions who make a contribution to the branching percentage of at least 3% at any reduced electric field value are included in the table and figure.

Below approximately 140 Td, the protonated parent is the dominant product ion observed for all ketones. This is in reasonable agreement with other PTR-MS studies. For example, in the study by Buhr et al. (2002) at one reduced electric field of approximately 140 Td, the authors showed that proton transfer from H_3_O^+^ to ketones will predominantly be non-dissociative, regardless of chain length. This limited dissociation observed in PTR-MS for reduced electric fields below 140 Td also agrees with studies using the thermalized conditions in Selected Ion Flow Tube—Mass Spectrometry (SIFT-MS) (Spanel et al., 1997; Smith et al., 2003, 2019), and suprathermal Selected Ion Flow Drift Tube (SIFDT) investigations (Specyvyi et al., 2017).

Above 140 Td, fragmentation of the protonated parent is observed, a fact that was not reported by Buhr et al. for 2-butanone, 2-hexanone, 2-heptanone, 3-heptanone, 4-heptanone, 3-octanone, 2-nonanone, and 2-decanone, for which only the protonated parent is observed. Limited fragmentation is, however, reported by Buhr et al. at 140 Td for 2-pentanone, with a product ion being observed at *m/z* 45, which we also observe and assign it to be C_2_H_5_O^+^ (protonated acetaldehyde) although it is found with a much higher relative intensity compared to the protonated parent in our study than found by Buhr et al. This difference in intensity is most probably associated with differences in the transmission of ions, because Buhr et al. used a quadrupole mass spectrometer.

In the present study, significant percentages of hydrocarbon ions, C_n_${\text{H}}_{\text{m}}^{+}$, are seen. This agrees with another *E/N* study of the ketones, 2-butanone, 2-pentanone, 2-hexanone, 2-heptanone, and cyclohexanone by Pan et al. (2017), who used a dipolar proton transfer reaction (quadrupole) mass spectrometer. Their study, which covered the reduced electric fields of approximately 50–110 Td, reported the *m/z* values of the product ions we have found, but observed substantially more fragmentation than we detected, even at their low *E/N* values. The amount of fragmentation reported at low *E/N* (as low as 50 Td) by Pan et al. is surprising, given that at these *E/N* values the reagent ion signal in our instruments would be protonated water clusters. This again illustrates that care must be taken when comparing results from different PTR-MS instruments.

In our study, typically 2–7 fragmentation channels have been observed. However, many of them were significant only at higher reduced electric field values. For instance, C_3_${\text{H}}_{3}^{+}$ and C_3_${\text{H}}_{5}^{+}$ ions occur only for *E/N* values higher than about 150 Td. Thus, for *E/N* values up to about 130 Td, the protonated molecules are dominant having well-above 80% branching percentages associated with that channel. Interestingly, the highest number of fragmentation channels was noted for 3-hexanone (7 channels) and C7 ketones; 2-heptanone (5 channels), 3-heptanone (6 channels), 4-heptanone (5 channels), and 2-methyl-3-hexanone (6 channels). As expected, heavier ketones are found to fragment considerably less.

For several ketones, the proton transfer process is followed by the elimination of an H_2_O molecule leading to the observed hydrocarbon ions C_n_${\text{H}}_{2\text{n}-1}^{+}$. However, these channels have small associated branching percentages, and at higher values of the reduced electric field undergo further fragmentation.

The channel leading to the C_2_H_5_O^+^ ion is very abundant in fragmentation patterns of C5 and C6 ketones. Interestingly, the mass spectra of C8 and C9 ketones do not have oxygen-containing fragmentation channels.

High humidity reduces the fragmentation of ketones. Interestingly, this effect is most evident for the *E/N* values of 150–160 Td. For example, the abundance of the protonated parent ion of 2-pentanone under normal conditions for 150 Td is 47%; whereas, in humid air, it has a branching percentage of 69%. The analogous values for 3-nonanone are 48 and 79%, respectively. This interesting dependence can be attributed to the formation of considerable amounts of protonated water clusters, which can react with ketones of interest. Consequently, far less energy is available for fragmentation in such reactions than for those associated with H_3_O^+^. At the higher *E/N* values formation of water clusters is suppressed and, thereby, the positive effect of humidity on having reduced fragmentation is weakened. The general effect of the higher humidity is to shift the product ion branching percentage curves by approximately 20 Td to higher *E/N*.

**Figure 3:**
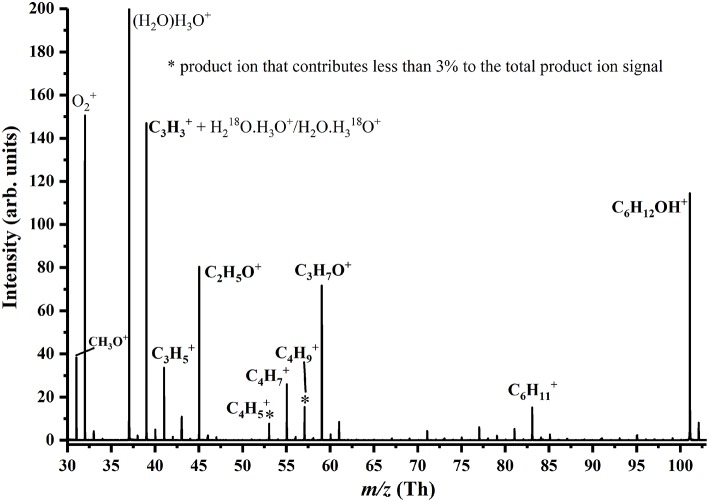
Mass spectrum for 3-hexanone recorded at 180 Td. Product ions coming from the compound are identified. The product ions C_4_${\text{H}}_{5}^{+}$ and C_4_${\text{H}}_{9}^{+}$ each contribute <3% to the total product ion percentage even at the highest reduced electric field investigated.

To illustrate the quality of the data, a mass spectrum recorded at 180 Td for 3-hexanone is provided in Figure 3. This highlights some product ions which are associated with the volatile, but are not included in the tabulation or Figure 2, because their contributions to the total relative abundance are <3% for any reduced electric field value.

## Conclusions

This work provides a large body of data and an extensive library of product ion distributions as a function of reduced electric field for the reactions of H_3_O^+^.(H_2_O)_n_ (*n* = 0 and 1) with a selection of ketones using the powerful analytical technique of PTR-ToF-MS. Although the study was originally conceived owing to the importance of ketones in the breath, and the need to determine what product ions should be monitored using PTR-MS, these results should be of interest to researchers working in other areas such as the environmental sciences and atmospheric chemistry.

A key outcome from this work is that product ion distributions at any specific reduced electric field can only be used to provide an indication of what ion-molecule channels are occurring. Detailed branching percentages are only specific to a given PTR-MS instrument and then under the specific operational conditions, not least the humidity present in the drift tube, as demonstrated in the results from this study.

Of the ketone isomers investigated in this study, it is apparent that it is not possible to provide any selectivity by manipulating the ion chemistry through changes in the reduced electric field. For this to be accomplished, the use fast gas chromatography coupled to PTR-MS is needed when analyzing gas samples that contain a mixture of ketone isomers, as often occurs in breath samples.

In the context of the ketones analyses in real breath samples by PTR-MS, the functional isomers of species from this chemical family (such as e.g., aldehydes) also need to be considered and investigated as their protonated forms cannot be separated from the respective protonated ketones. The ketones' PTR-MS analyses in the presence of their functional isomers require further studies. However, it is worth mentioning here, that aldehydes undergo significant fragmentation in the PTR-MS instruments and the abundance of their protonated parent ions is usually very small (<10%) (Buhr et al., 2002; Schwartz et al., 2009). Consequently, the presence of aldehydes in the breath sample can only have minor influence on the parent ions of the respective ketones.

## Data Availability

The raw data supporting the conclusions of this manuscript will be made available by the authors, without undue reservation, to any qualified researcher.

## Author Contributions

MM, DOL, and FP are Early Stage Researchers employed on the EU IMPACT ITN. They contributed equally to the experimental measurements, data analyses and contribution to the completion of this paper. PM proposed the study. PM, PS, SJ, CAM, and TDM contributed equally to the writing of the paper.

### Conflict of Interest Statement

FP and TDM was employed by company IONICON Analytik Gesellschaft m.b.H. The remaining authors declare that the research was conducted in the absence of any commercial or financial relationships that could be construed as a potential conflict of interest.
